# Automated Design and Integration of Asset Administration Shells in Components of Industry 4.0

**DOI:** 10.3390/s21062004

**Published:** 2021-03-12

**Authors:** Jakub Arm, Tomas Benesl, Petr Marcon, Zdenek Bradac, Tizian Schröder, Alexander Belyaev, Thomas Werner, Vlastimil Braun, Pavel Kamensky, Frantisek Zezulka, Christian Diedrich, Premysl Dohnal

**Affiliations:** 1 Faculty of Electrical Engineering and Communication, Brno University of Technology, 616 00 Brno, Czech Republic; arm@feec.vutbr.cz (J.A.); xbenes23@vutbr.cz (T.B.); bradac@feec.vutbr.cz (Z.B.); zezulka@feec.vutbr.cz (F.Z.); dohnalp@feec.vutbr.cz (P.D.); 2 Institute for Automation Engineering, Otto von Guericke University Magdeburg, 39106 Magdeburg, Germany; tizian.schroeder@ovgu.de (T.S.); alexander.belyaev@ovgu.de (A.B.); thomas.werner@ovgu.de (T.W.); christian.diedrich@ovgu.de (C.D.); 3 Compas Robotics and Compas Automation, Nadrazni 610/26, 591 01 Zdar nad Sazavou, Czech Republic; vlastimil.braun@compas.cz (V.B.); pavel.kamensky@compas.cz (P.K.); 4 Department of Technical Studies, College of Polytechnics Jihlava, Tolsteho 1556, 586 01 Jihlava, Czech Republic

**Keywords:** Asset Administration Shell, digital twin, Internet of Things, industrial Internet of Things, Industry 4.0, Manufacturing Execution System, Manufacturing Operation Management, Open Platform Communication—Unified Architecture (OPC-UA), MQTT

## Abstract

One of the central concepts in the principles of Industry 4.0 relates to the methodology for designing and implementing the digital shell of the manufacturing process components. This concept, the Asset Administration Shell (AAS), embodies a systematically formed, standardized data envelope of a concrete component within Industry 4.0. The paper discusses the AAS in terms of its structure, its components, the sub-models that form a substantial part of the shell’s content, and its communication protocols (Open Platform Communication—Unified Architecture (OPC UA) and MQTT) or SW interfaces enabling vertical and horizontal communication to involve other components and levels of management systems. Using a case study of a virtual assembly line that integrates AASs into the technological process, the authors present a comprehensive analysis centered on forming AASs for individual components. In the given context, the manual AAS creation mode exploiting framework-based automated generation, which forms the AAS via a configuration wizard, is assessed. Another outcome consists of the activation of a virtual assembly line connected to real AASs, a step that allows us verify the properties of the distributed manufacturing management. Moreover, a discrete event system was modeled for the case study, enabling the effective application of the Industry 4.0 solution.

## 1. Introduction

The concept of Industry 4.0 (I4.0) has been investigated and developed in economically advanced countries for at least 5 years [1,2,3,4,5,6]. In this context, the most important research groups include ZVEI, VDI/VDE, and BITCOM, especially in terms of refining models such as Reference Architectural Model Industrie 4.0 (RAMI 4.0) and, consequently, the I4.0 component model [7,8]. The entire strategy gradually evolved in Germany and spread across Europe. In 2018, three European countries began to collaborate closely within the manufacturing domain to improve and disseminate the concept, and their efforts yielded the following initiatives: the Alliance Industrie du Futur in France, the German-based Platform Industrie 4.0, and the Piano Industria 4.0 in Italy [9]. These actions and policies enabled innovative ideas to expand into other domains, such as standardization, industrial communication [10,11], informatics [12,13,14], functional safety, cybersecurity, economics, marketing, energy production, and social economy. Most notably, the diversity of influences has been reflected in the concept of smart factories [15,16,17,18,19,20]. Outside Europe, the scheme has found wide reception in the USA, China, and Japan.

Implementing the principles of I4.0 into industrial applications is a slow process, mainly due to the generally nonsystematic approach. At present, relevant technologies involve and rely on digitization, robotics, non-optimal data acquisition, virtual reality, IoT, and advanced data processing [21,22,23,24,25,26,27]; simultaneously, however, application standards remain undeveloped or are lacking completely, and a similar deficiency also affects corporate economy and common initiative in any given field [28,29,30,31,32]. Conversely, these separate technologies help to accelerate the implementation of I4.0 principles and open new opportunities and challenges for technical development; in the given context, such benefits were considered unfeasible 5–7 years ago. The overall impact of I4.0 and its recent transformations or outcomes—digitization and virtualization in particular—can then be interpreted as epitomizing the difference between the present situation and the conditions preceding the introduction of the initial I4.0 in 2013.

In the current process control, the Manufacturing Execution System (MES) and Manufacturing Operation Management (MOM) play integral roles as the central points of job planning and management [33]. Thus, all relevant data must be transferred to these software, of which only the MES can execute a job command task. Conversely, the concept of I4.0 relies on decentralized (distributed) control—i.e., procedures without a central entity; the decision-making process is then distributed between the entities in the communication network. Within this concept, the MES/MOM ensure new product initiation and are not involved in the job scheduling stage.

A major component of I4.0 is embodied in the AAS, which, in the industrial domain, characterizes assets such as the product, machine, equipment, and factory; an AAS also communicates with other AASs as standard entities interconnected throughout a network. The actual concept originates from a novel interpretation of the management, where relevant components are integrated both horizontally and vertically. While the current management methods are structured mostly vertically, in a hierarchical manner, the novel approaches exploit the markedly higher intelligence (managing capabilities) of the individual manufacturing components, from the top level items down to the sensors and actuators. This concept changes the architecture of the industrial process control system into a distributed (decentralized) form, embedding flexibility in job scheduling, failure responses, and product customization.

The authors characterize a novel procedure for the automated creation of AASs via a configuration wizard, the aim being to accelerate the formation process and to achieve the easier implementation of AASs. In functional terms, the administration shells are generated in compliance with the requirements and standards of I4.0. The operability of the design is verified on a case study involving an assembly line to produce printed 3D toy cars; this step also comprises considering and comparing two communication protocols, Message Queuing Telemetry Transport (MQTT) and Open Platform Communication—Unified Architecture (OPC UA).

This paper discusses AASs (Chapter 2) together with a methodology for creating the wizard; this methodology is based on requirements relating to the functionality, formation, and structure of the AAS. The virtual production testbed and implementation are partially analyzed in Chapter 3, which also defines the communication interface separating the administration shell from the asset; in our case, the assets embody the virtual manufacturing components and items that participate in the manufacturing procedures. The results, outlined in Chapter 4, are characterized more broadly in the last section of the article, with relevant research perspectives complementing the overall discussion of the project (Chapter 5).

## 2. Asset Administration Shell

The Asset Administration Shell (AAS) is a major constituent of I4.0, creating an interface between the physical and the virtual production variants. An AAS represents—virtually, digitally, and actively—an I4.0 component in the I4.0 system. Any production component in the I4.0 environment has to have an administrative shell [34,35,36,37,38].

In addition to multiple other modes of use, the AAS facilitates the virtualization of the manufacturing process to model, fine-tune, and monitor the algorithms and economy of production already before the cycle actually starts [39]. The AAS is an indispensable precondition for decentralized industrial manufacturing management, yielding flexibility and emergency robustness to reduce queues, bottlenecks, and other issues that limit the efficiency of production units during their service lives.

Alternatively, the AAS can be also designated as the digital twin of a production component [40]; in this context, however, it has to be emphasized that our approach strictly observes and exploits the rules or procedural laws presented in the literature [7,8,9,10].

**Figure 1:**
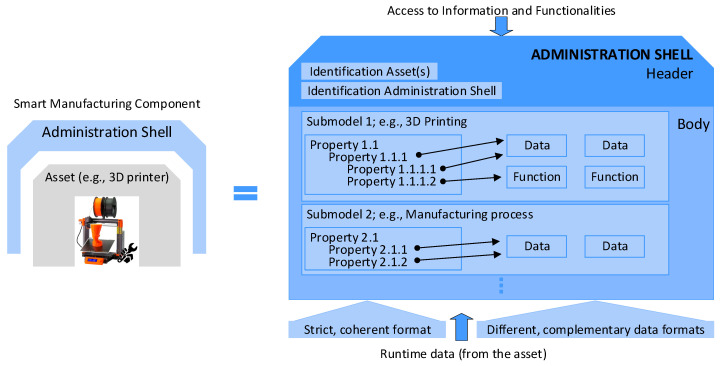
The detailed structure of an AAS.

Figure 1 shows the structure of and connection between a physical item and the corresponding administration shell (AS). A component within I4.0 integrates an asset and its electronic model—i.e., the appropriate AS. The AAS in Figure 1 consists of a body and a header. The header contains identifying details regarding the AAS and the represented asset, and the body comprises a certain number of submodels to facilitate the asset-specific characterization of the AAS (see [41,42,43,44,45,46,47,48]).

The submodels represent different aspects of an asset. Possible aspects and associated submodels encompass, among others, the following items: identification, communication, engineering, configuration, safety, security, lifecycle status, energy efficiency, and condition monitoring.

Each submodel contains a structured quantity of properties that can refer to data and functions. The properties are specifiable in accordance with the standard IEC 61360, but the data and functions can be defined in various formats. Figure 1 shows a graphical example of an AAS [7].

**Figure 2:**
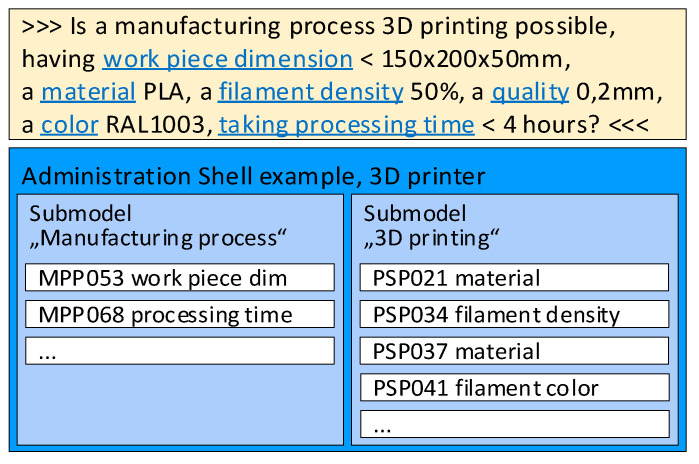
The bidding process related to specific submodels of the AAS.

The bidding between two assets on an industrial assembly line consisting of 3D printers is described in Figure 2, where an asset (such as a semi-finished product) asks another asset (a 3D printer) on the assembly line if its capacity, functionality, and availability can ensure the completion of the task using the pre-specified parameters (for example, the dimensions of a printable semi-finished product must not exceed 150 × 200 × 50 mm; the applied material is PLA with a filament density of 50%; the color corresponds to RAL1003; the layer thickness equals 0.2 mm; and the printing time has to be below 4 h).

The requirements concerning the contents of AASs can be classified into three groups [7,9]:

1. General;
2. identifier-related;
3. AAS-specific.

**Figure 3:**
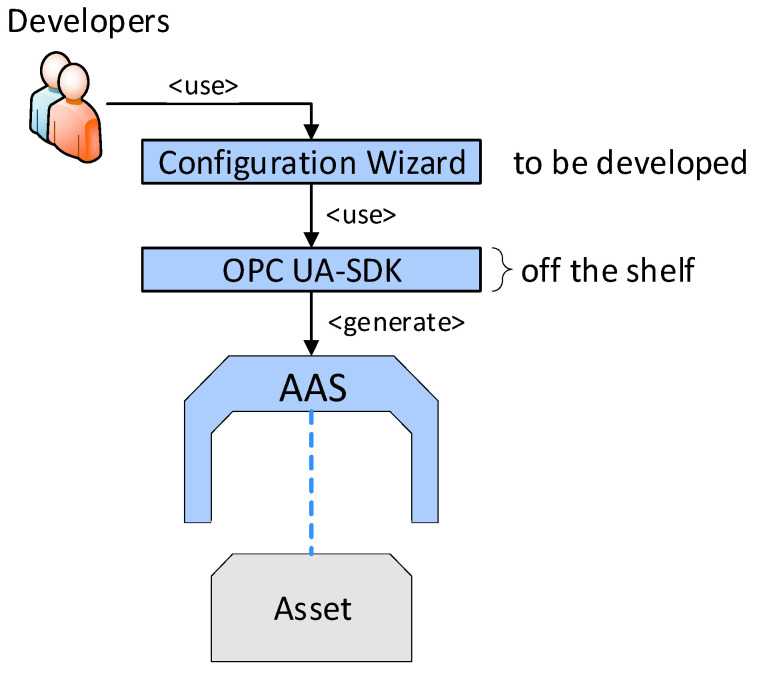
A block diagram to define the functioning of ConfigWizard.

All such requirements are specified in sources [7,9] and included in our proposal. Exploiting knowledge of the procedural principles relating to AASs and their practical usage, we designed ConfigWizard, an innovative tool to allow the comfortable and partially automated generation of AASs. To fulfill this purpose, the software assists in the essential steps that enable AAS formation and functions (access via a webservice; information modeling: submodels, parameters, and events; asset integration: the mapping of the communication properties; OPC UA server configuration), see Figure 3.

**Figure 4:**
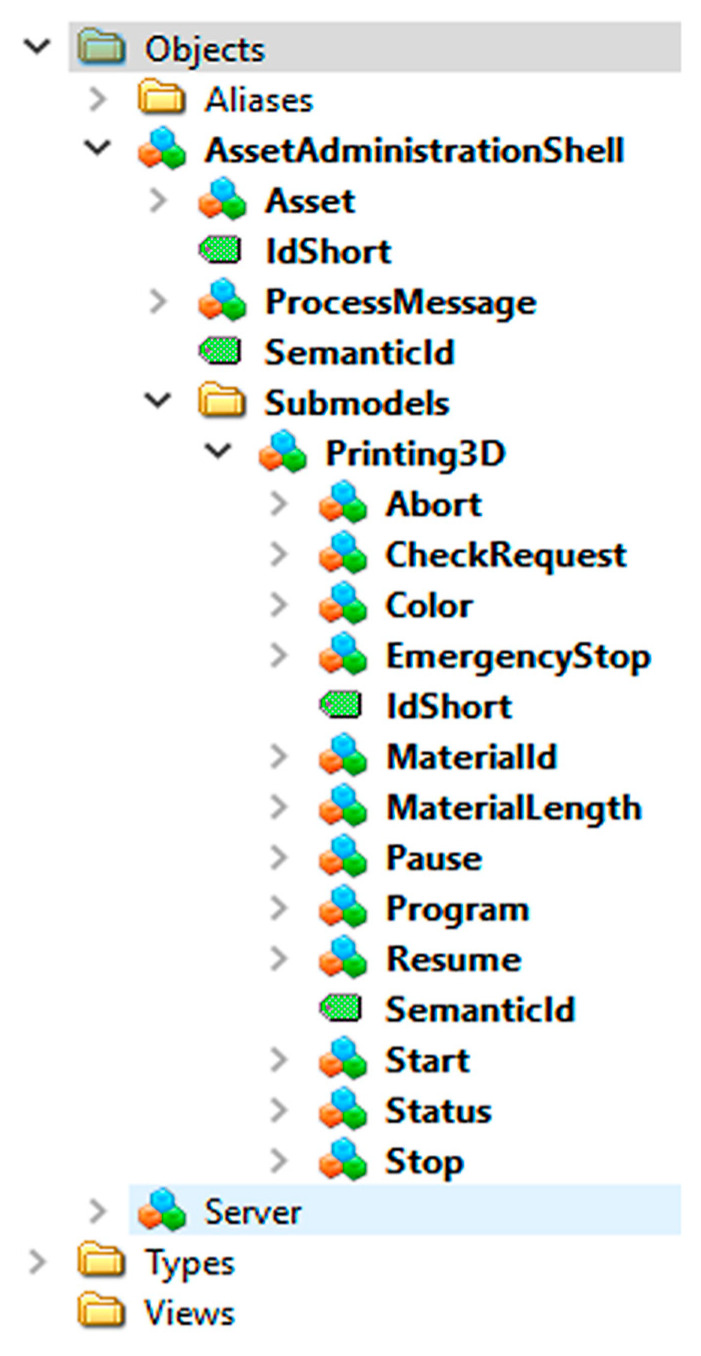
A manually formed AAS.

**Figure 5:**
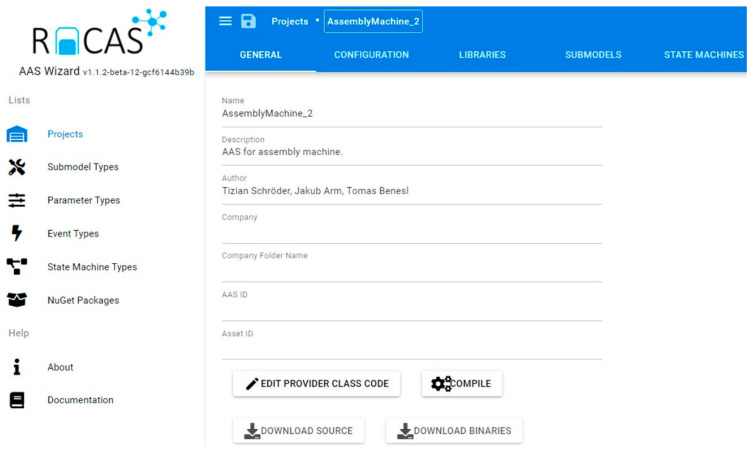
A ConfigWizard screenshot: front end.

Without such a configuration wizard, all the steps must be carried out manually, requiring intensive programming, see Figure 4. The ConfigWizard reduces the AAS development efforts to inserting relevant configuration data via a GUI (frontend, Figure 5). The user can add, edit, or delete each of the AAS submodel entities, such as a property, method, or event. The ConfigWizard’s backend then automatically generates an AAS software package based on the configuration entered by the developer; the necessary configuration data are usually derived from a scenario-specific use case and sequence diagrams.

Regarding the underlying OPC UA technology [49,50,51,52,53,54,55,56,57], the user must also define the parameters of the OPC UA channel and other items according to the OPC UA stack—i.e., in agreement with the OPC UA standard at each level of the ISO/OSI model (Table 1). Using this procedural step, the connection with the AAS environment is established by the OPC UA.

ConfigWizard thus allows us to avoid accessing the OPC UA server creator (our research relied on Unified Automation) itself; instead, it facilitates the utilization of a user-friendly, web-based wizard. The most significant advantage of the tool consists in the ability to create the OPC UA nodes automatically, especially if there are more objects of the same type (for example, more temperature sensors in a machine unit). In terms of the fundamental idea, development, and testing, the Wizard for the automatic configuration of AASs in different assets fully exploits the long-term experience of the authors of this paper, offering two ways to implement I4.0 components:

- Manually formed AASs (indicated in the Industry 4.0 component model, Figure 4).
- Automated AASs (see ConfigWizard, Figure 5).

## 3. Implementing the Industry 4.0 Component Model

**Figure 6:**
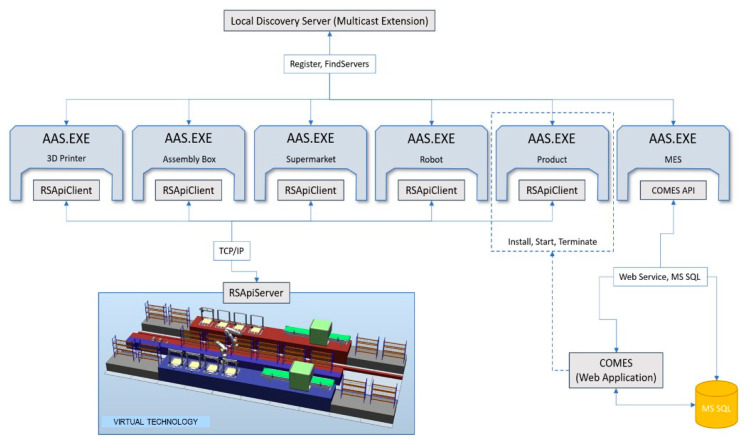
The architecture of the presented case study.

**Figure 7:**
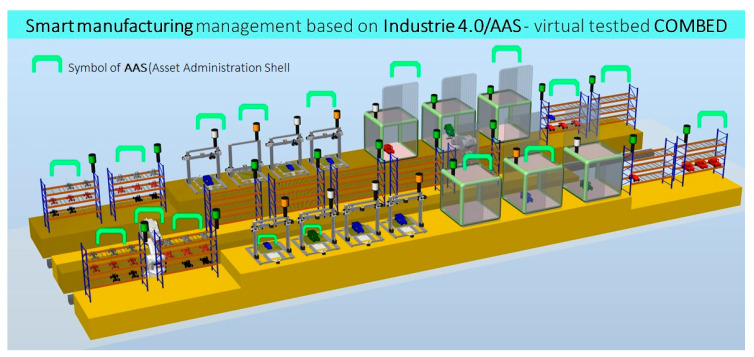
The virtual production segment (COMBED) introduced at the 2019 International Engineering Fair in Brno, the Czech Republic.

This chapter discusses the procedures, standards, programming languages, communication methods, interfaces, bidding processes, and all associated elements that are necessary for the successful realization of the “factory of the future”. This case study demonstrates the use of ASSs in an I4.0 virtual assembly line designed to produce plastic models of cars (Figure 6 and Figure 7).

### 3.1. Case Study

The case study is based on a virtual production technology (the COMBED virtual testbed), as shown in Figure 7, consisting of two assembly lines with assets—i.e., machines (3D printers, assembly boxes), transport robots, and storage racks. The study demonstrates a smart production management method which utilizes smart assets according to the I4.0-based component model. Each virtual asset (for example, a product, machine, robot, conveyor, line, or warehouse rack) has its administration shell. The AASs communicate with each other and negotiate the production priorities and requirements according to a pre-specified set of rules. The manufacturing operations are negotiated by a product with respect to the principles of I4.0, enabling us to incorporate smart features into the production processes.

The COMBED system is employed to demonstrate the automated optimization, adaptation, and setup on an example of a production segment that manufactures products to order. Multiple scenarios are possible and can be adapted by the user, in view of the tables of parameters; the options either consider the “ideal” state or assume failures and downtimes to approach practical conditions. Based on these scenarios, we can test the smart production management’s responses to diverse situations in real-world industrial cycles. Our solution automatically modifies the product processing steps and stages (material flow) to allow the use of currently available tools. The manufacturing management is also capable of supporting very flexible production cycles (in small orders—i.e., ones down to batch size 1), as it automatically and in real time adapts the equipment to the manufacturing operations required by the product variant or specifications (auto-setup). With flexible machinery, the factory can simultaneously manufacture various products and their versions, and the equipment setup operations eliminate the losses that otherwise accompany the material/semi-product transport. The case study utilizes COMBED to demonstrate the manufacturing of simple products—namely, plastic toy cars, each comprising a body and a chassis.

Our smart production management technique features a completely new, decentralized approach using the ideas and standards of the Industry 4.0 platform. The actual research involved applying and refining some of the objectives of I4.0, including automated optimization, adaptation, and setup of the manufacturing and logistics equipment; all of these steps were performed according to the needs of the manufacturing operations required by the product, as also stipulated within I4.0. Importantly, the entire project was designed with respect to observing the possibilities and benefits provided by the Plug and Produce (P&P) option. This mode enables machine builders to deliver their technologies with standardized AASs, allowing factories that run P&P to smoothly incorporate a new asset into the product negotiation process. The new asset carries its features, abilities, and parameters in the AAS submodels, facilitating the smart production management process.

### 3.2. Production Control Function of the AAS

**Figure 8:**
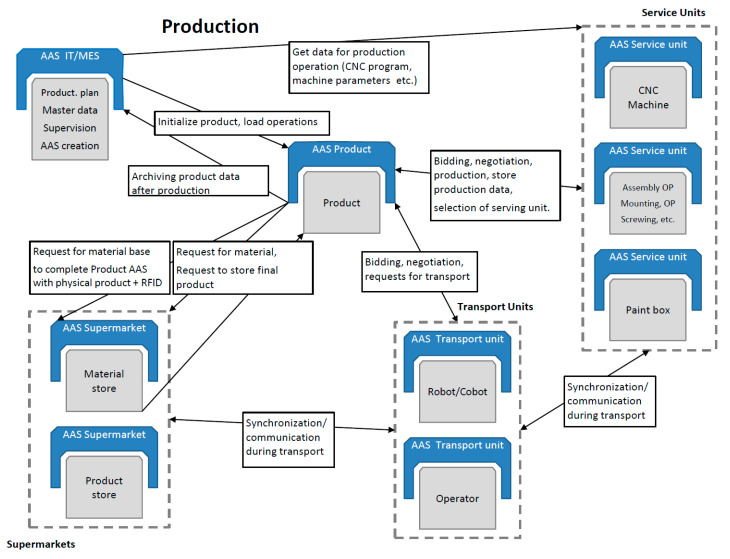
The data flow in a smart factory.

**Figure 9:**
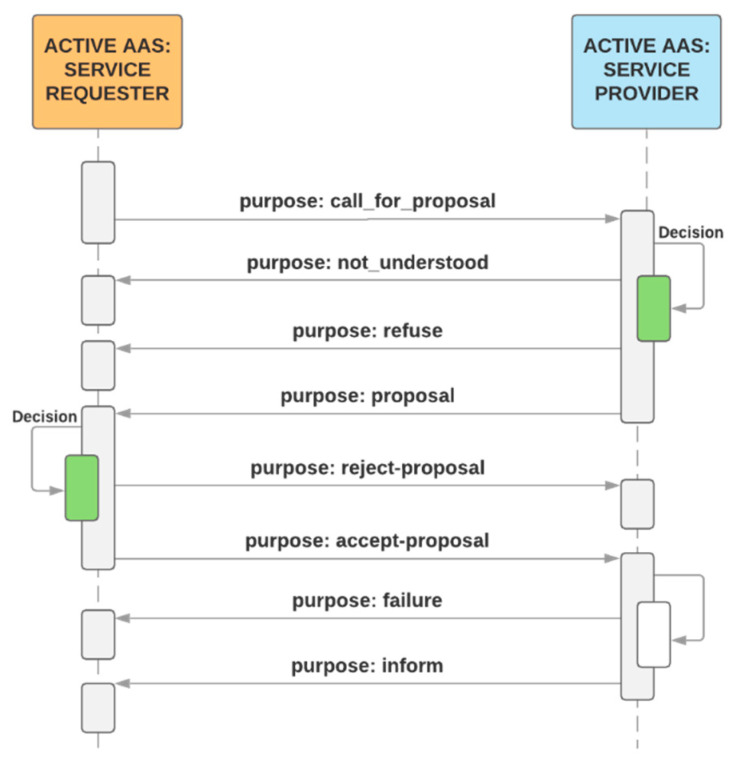
The bidding sequence.

With the scenarios (meaning production scenarios that simulate manufacturing behavior at various limit states), the smart production management can be tested and easily evaluated by standard MESs, as are often applied in factories. The MES is routinely employed to compute manufacturing efficiency and other relevant indicators, and an interconnection between this system and the AAS would allow the computing functions to be suitably utilized and expanded. For such evaluation of the management, we used the COMES MES/MOM system, collecting data from the COMBED virtual assets to validate the KPI (downtime analysis, Overall Equipment Effectiveness—OEE, and other relevant indicators). In a real-world factory, this approach is expected to yield innovative effects, including automatic production control according to the objectives pre-specified by the factory managers (for example, in response to the market situation) and high robustness of the manufacturing processes, which thus resist diverse types of failures. From the perspective of production control, the AAS functions can be classified into 3 implementation groups, as follows: a service requester (SR), a service provider (SP), and a common part of the code, involving such operations as communication and logging. Together with structured access to data, negotiation embodies a key AAS functionality. To ensure appropriate control, it is important that each SP be able to offer its services. The SR can browse through the SP to find a service ideal for the processing of the required operation. Figure 8 and Figure 9 indicate that products actually are SRs that negotiate tasks to secure their own production.

However, manufacturing units, such as a CNC machine or an assembly line, require service intervention, material, tools, maintenance, and other steps or items; in such situations, the units become SRs to negotiate their requirements. Thus, the negotiation submodel has to be fully implemented in each AAS.

**Figure 10:**
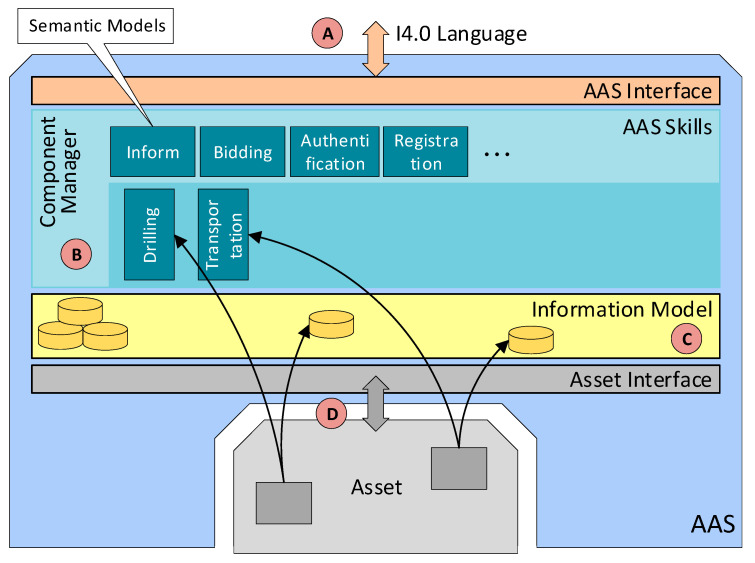
A block diagram of an AAS.

Figure 9 illustrates the standard negotiation sequence applicable to any operation. This sequence embodies an automated process comprising a demand, offer (call for proposal), order (proposal), and confirmation. With the algorithm, it is possible to request all available SPs offering services and select the most suitable SP. The discussed actions and processes then create the theoretical area that enables us to investigate, implement, and improve the optimization algorithms, exploiting, for instance, the condition where a demand is not valid only for the next manufacturing step but facilitates negotiating all the production stages, including transport. In implementing the wizard-formed AASs, the basic content element is the Component Manager (part B in the Figure 10), which brings together the sub-models to support the functionality of the AASs. The SR negotiation algorithm begins with the requirement for another component—namely, the mode in that no production step is active or scheduled for the product and the production unit does not need any service operation or resources. The Component Manager initiates negotiation to create a Call for Proposal (CfP), which is passed on to the Interaction Manager (IM), and the IM then sends the CfP to the service-supporting device. The communication between the individual AASs utilizes the OPC UA communication protocol, allowing the messages to be sent in the JSON format. The OPC UA framework alone interacts with the lower layers of the ISO/OSI model, requiring the user to implement the application layer only (Figure 6). The data in the JSON format are well readable and ideal for debugging the algorithms and testing the functionality; in future aggregations, a lower data size message format will be applicable if necessary. When the waiting time for the offers has expired, the IM will pass on the proposals available, and the negotiation algorithm will call the optimization function to select the best bid. Subsequently, an order is created and handed over to the IM, the SP confirms the order, and the negotiation of the next production step terminates.

Due to the concurrent communication, the SP may encounter a situation where more than one proposal has to be responded to before being accepted by the SR. We suggest that the problem be resolved via one of the following approaches (for illustration, we selected the first option):

1. The SP will not respond to any other CfP before an acceptance or rejection is received. This scenario involves ineffective communication arising from the undefined busy time of the SP.
2. The SP will add the SR (sending the CfP) to a queue; if accepted, the SR’s CfP will be handled by using one of the queue’s algorithms (e.g., first come, first served). Moreover, the SP could inform other SRs to cancel the request.
3. The SP will add the SR (sending the CfP) to a list; if accepted, the SR will be selected by the pre-defined priority and other SRs will be informed of the delay.

The manufacturing commands are based on the PackML standard. The product, if on the requested spot, sends the “Start” command to change the production unit’s status according to the current stage of the manufacturing cycle. At the end of the cycle, the status signal “Done” appears to complete the current production phase. The negotiation and transport are carried out until the final product has been located in the warehouse or another outgoing point. The production process requirements for the SRs should be defined in the CfPs, including whether the relevant data are to be retained by the production unit’s AAS or deleted after negotiation. If the data are not to be retained, the SR will send them again before the start of the manufacturing cycle. In the current implementation of our AAS, the data are sent out immediately before the “Start” command; it would nevertheless be more advantageous if the production unit’s AAS stored the CfPs’ data, mainly due to the busy communication lines in larger-scale production. The hypothetical scenario, however, places greater demands on the AAS’s data storage space in the case of long-term production planning.

### 3.3. Interating the AASs into the Demonstrator

**Figure 11:**
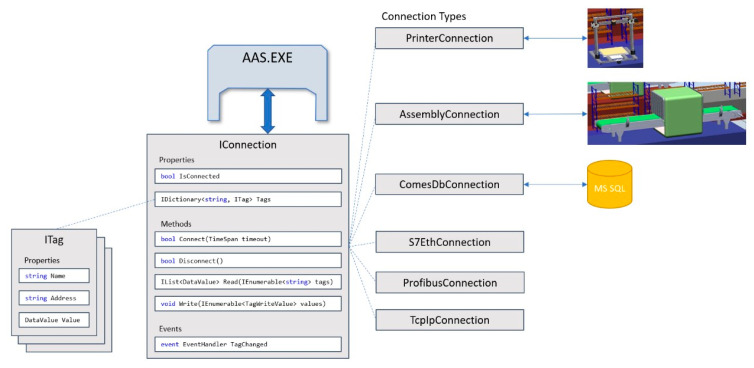
Integration of different communication drivers without rebuilding the AAS.

The COMBED system, characterized in the previous chapter, replaces the real assets (production machines) in the factory. The simulation tool facilitates integrating a “Smart Component” that behaves like a server. A client-server connection is then established for each device. The client simulates a control system, such as a programmable logic controller (PLC), and runs independently of the AAS, requiring the designer to create a communication interface between the asset (client) and the administration shell (Figure 11). This communication interface is formed as a tag definition, which can be sent to the asset. In our implementation, the AAS communication driver integrates a TCP/IP connection and sends a TCP stream; thus, it is possible to employ any communication protocol and simply assign it to the selected AAS.

The AAS design, whose implementation allows using any communication driver for diverse types of assets, is indicated in Figure 10, part D. However, we have to follow the standard for communication with I4.0 components via the I4.0 language (part A in Figure 10). Figure 11 presents in detail the integration of different communication drivers without rebuilding the AAS or submodels. The tags are created by using the ITag definition, which needs to be linked to an asset—i.e., a control PLC, a distributed control system (DCS), a database, or another component.

In the given context, Read and Write methods must be implemented to enable data exchange. If the AAS hardware is able to use not only Ethernet but also other interfaces (RS485/232), we can establish communication with almost any asset. The overall implementation of our AASs is carried out in C#, using NET Core to ensure platform independence. However, there may appear a difficulty with the OPC Foundation’s local discovery server (LDS), as this server can be installed on Windows only. In general terms, using AASs on embedded devices or single-board PCs such as the R-Pi requires a Global Discovery server or a different implementation of the LDS server. During the testing, MQTT-based communication was also employed, exhibiting communication latencies lower than those achieved by the OPC UA; in the MQTT option, however, a centralized broker had to be utilized. Such an approach appeared to suit both the fine-tuning of the algorithms and the whole scenario. In real-world applications, the OPC UA technology is more convenient than MQTT because, thanks to the LDS Multicast Extension, it can be used without the central element (broker). An administration shell is formable manually by such steps as providing data structures, tags, and other elements during the actual development and implementation phases; however, to simplify the generation and configuration, the wizard characterized in the previous chapter has been developed.

### 3.4. Formal Modeling

**Figure 12:**
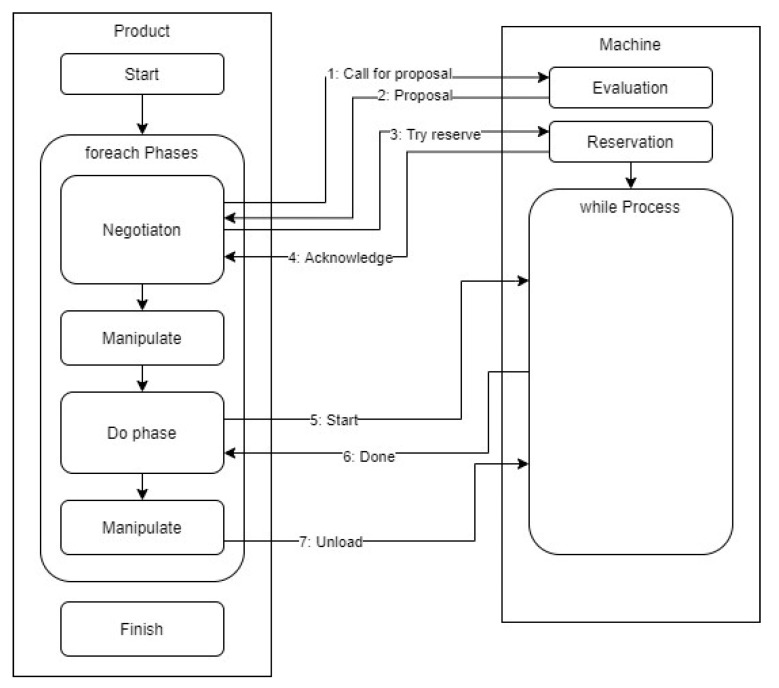
The architecture and flow of the discrete event simulation scenario.

In addition to the continuous-variable dynamic simulation, as outlined above, we also created a formal model using the discrete event system technique (powered by the SimPy library available in Python). This model reflects our use case and consists of entities such as a machine and a product (the simulation design is depicted in Figure 12). Utilizing these elements, we follow the command level of details; thus, every machine or product can interact with the others via commands (such as the call for proposal, start, and unload) and events (such as started, production phase done, and unloaded). In this context, the modeled production then comprises the bidding sequence and the Pack-ML interaction concept.

Moreover, fault injection is incorporated into the simulation, allowing us to induce a failure in the machine operation phase and, thus, to simulate downtimes. In failure activation, based on the assumed exponential time distribution, the machine changes its state, informs the product, and waits a Gaussian time to facilitate the repair cycle. Meanwhile, the product aborts the current operation to launch the negotiation routine, attempting to find another machine to be served. To transform the simulation code into a discrete event system, some issues must be mitigated. The relevant tasks include decomposing the execution code into atomic chunks according to the discrete event system definition; in the bidding interaction, adopting the separate service (machine) reservation technique instead of reservation during CfP handling; and ensuring that the service proposal evaluation is atomic across all the machines in the factory that are associated with the product. The discrete event simulation can run under various conditions and settings. Thus, the machine counts and operation times were specified as close as possible to the dynamic simulation settings, and we incorporated the Gaussian time in every operation (manipulating, producing). Unlike the dynamic demonstrator, we simulated random product initiation (one product per 2 s) and applied different failure-injection procedure settings.

## 4. Results

To compare the manual (via an OPC client) and automated (utilizing the presented wizard, Figure 5) approaches to the formation of an AAS, we identified the pros and cons qualitatively (Table 2).

The wizard-based designing was tested on the COMBED testbed, which contains several machines involved in the manufacturing cycle. Each of these units is autonomous and has an AAS capable of negotiating with other AASs. A product entering the cycle asks for the services that allow it to be produced, and the machine is selected according to the price and relevant associated parameters, with respect to the prespecified optimization criterion. Importantly, the position of the machine on the assembly line is a major factor determining how the products will be transported during the operational stages and after completion, namely, when they are to be handed over to a distribution point or warehouse. The advantage of autonomous machinery consists in its quick response to a failure. In the event of a fault, the affected machine switches to the non-service state; if the problem persists, the product can be re-routed and negotiated with another machine. After being repaired, the machine returns to the operating mode to start offering its services again. At this point, the unit may alter the price of the services due to the increased OEE. The testing involved fourteen machines and nine warehouse rooms, with diverse quantities of products entering production at different moments; importantly, the scenarios also comprised failure and repair times. The entire simulation cycle was conceived to determine whether the AAS product algorithms can respond to emergencies, normal failures, and similar states or conditions. In all of the scenarios tested, the planned products were manufactured without operator intervention, as is typical of an ideal operating scheme. Regarding the communication latencies, with a larger number of one-minute assets (the OPC UA clients and servers) the delays were so long that the timeouts expired.

Initially, the tests were performed on only one PC, which hosted all of the AAS instances. In this operation, the communication issues were not as prominent as those that accompanied the scenario utilizing 14 computers with routers and switches, because the local host interaction did not involve major packet delays, eliminating retransmission. The high latency rates were primarily caused by the firewall and persisted even after deactivation; due to this fact, MQTT replaced OPC UA, resulting in a significantly lower latency. Considering possible origins of the issue, the OPC UA’s inferior performance may have been induced by a bug in the applied framework. The latencies ranged from hundreds of milliseconds in OPC UA to tens of milliseconds in MQTT; see Table 3.

**Figure 13:**
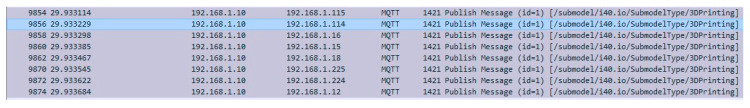
The CfPs from the products to the 3D printers.

**Figure 14:**
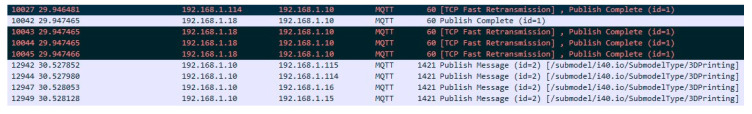
The retransmission of unacknowledged messages and new calls for proposal.

Using MQTT in a network of multiple PCs is associated with certain problems, and these affected the AAS testing cycles on some of the computers. Generally, the issues manifested themselves as follows: When initiated, an AAS product began to actively negotiate the first service (Figure 13). This service, however, was being simultaneously targeted by multiple other products, rendering the machines’ AASs unable to respond quickly enough; thus, after the timeout has expired, the product started to renegotiate the required item, and the collision domain became congested almost immediately. The firewalls, switches, routers, and related network components then caused spurious competition between the messages and, consequently, their erroneous processing (Figure 14). As the response time was found to be within units of seconds, a timeout would have had to equal at least 10 s; such an approach, however, might eventually lead to undesired delays in the manufacturing cycle.

With multiple devices in the network, OPC UA appears to be more beneficial, especially if installed together with local discovery servers (LDSs) and supported by a multicast extension (ME). This architecture, however, requires swapping the public keys (the PKI standard); in such a procedure, each device to be registered by the LDS server provides its public key, thus becoming a trusted item. In large networks comprising multiple LDS servers, however, the same problem as that affecting the use of MQTT may appear.

The testing and measurement cycles allow us to conclude that MQTT does not match conveniently with a greater number of AASs; in this context, OPC UA embodies the more suitable option, despite the demanding implementation and the necessity of transferring the public keys.

**Figure 15:**
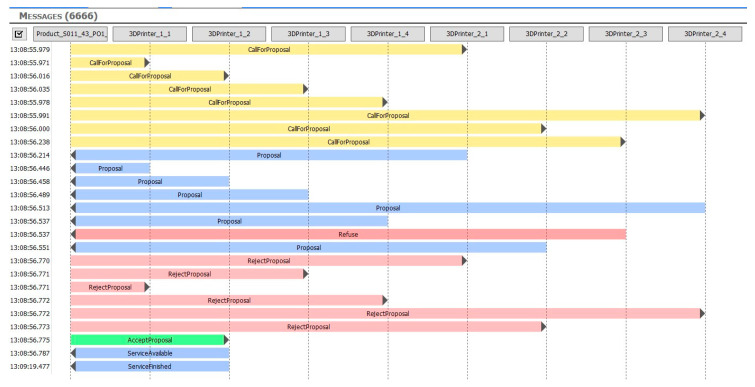
The bidding between a product and multiple printers.

To test and fine-tune the algorithms, we created an environment to visualize the messages sent between the individual assets. This procedure enabled us to define the communication latency and the number of messages required to complete the test scenarios. The bidding process messages are presented in Figure 15.

**Figure 16:**
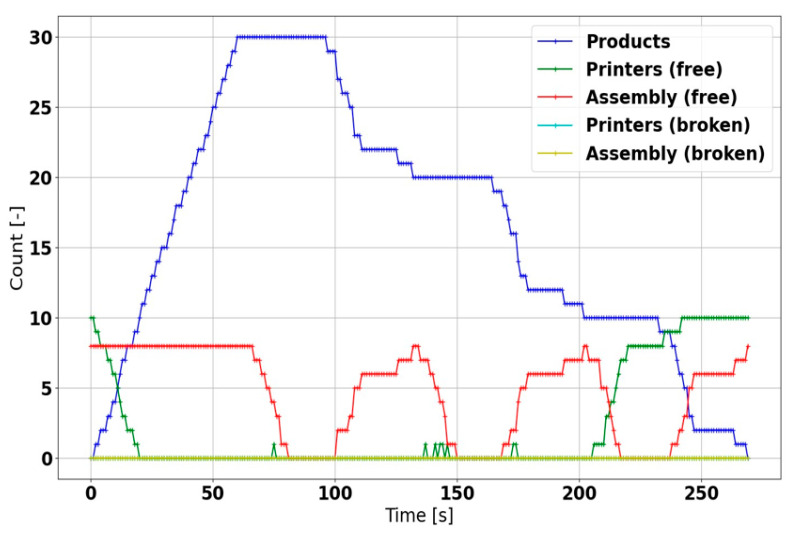
The regular discrete event simulation scenario.

**Figure 17:**
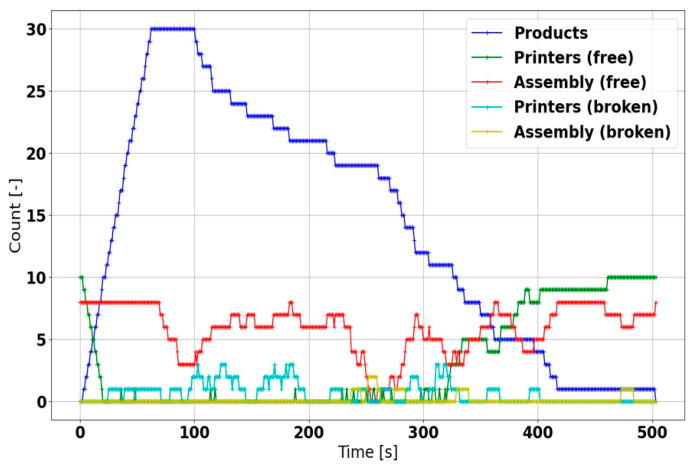
The discrete event simulation scenario with failure injection activated.

Regarding the discrete event simulation, the results also indicate that the manufacturing cycle is capable of fulfilling the product requirements as fast as possible in normal conditions (Figure 16); with multiple products to be served, however, the availability of free machines becomes markedly reduced. Moreover, in a machine failure, the product operations are actively restarted without any intervention from the central system, ensuring the completion of all products; the overall production time nevertheless increases (Figure 17).

The entire procedure, comprising 30 products, took 1.94 s using a single-threaded engine on a normal PC and covered about 300 s of the manufacturing cycle. The following run was characterized by the simulation time span of 31.365 s, and the computational time equaled 15.749 s. Thus, the results exhibited a strong correlation between the simulation and execution times, an effect that could be caused by the large amount of short-term events.

## 5. Discussion and Conclusions

The results show that the ConfigWizard software allows an AAS to be formed in a clearer and more user-friendly manner, especially as regards the specifications of the individual submodels and their parameters, methods, and events. The automated AAS generation process then not only saves a substantial amount of time but also utilizes relevant standards according to the user-defined input data, such as the names and attributes of the parameters. Another outcome of the research consists of exploiting the generated AASs to create a virtual manufacturing demonstrator facilitating production management. The interface between the AAS and the actual assets of the virtual demonstrator can be characterized already at the stage of designing the individual AASs, via both parameterizing the communication technology and mapping the transmitted variables. The interface of the real asset is then specifiable in the same manner. Within the presented use case, the production management utilizes AASs that comprise functions outlined in distributed production planning as set out through I4.0—namely, functions to enable bidding between semi-finished products which require processing services and also between machines or tools providing such services. The initial simulations (both the dynamic and the event systems) indicated that the manufacturing system flexibly responds to incoming requirements for new products (by including them in the queue) and actively resolves problems associated with manufacturing faults. To optimize the applied distributed production planning, it is, however, necessary to perform multiple simulations, all complemented with artificial intelligence algorithms. For this purpose, the created event- system simulation is considered the best candidate. The dynamic simulation, namely, the integration of the AASs in the test demonstrator, yielded the manufacturing times needed to produce the virtual car. A more significant parameter nevertheless lies in the service bidding mean time (the period required to accept or decline a bid), which, in the described scenario (5 AASs in a local network), ranged within lower hundreds of milliseconds. The outcomes of the discrete event simulation point to the suitability of a short time horizon and the need of an engine optimization process. In order to be usable by machine learning algorithms, the model should work as fast as possible to support a high amount of simulation iterations; this paper then proposes a convenient trade-off between the complexity and quick executability of the model to maintain the functions sufficiently credible. A major factor supporting smooth applicability of the system and related procedures can be identified in the fact that the AAS actually performs its functions, using a standard communication interface to operate in the heterogeneous environment of a manufacturing plant. The AAS is present at all levels of automated plant management, facilitating their effective interconnection. Thanks to the standardized parameters, attributes, events, and communication, the data associated with the design, preparation, order, and manufacturing stages are eventually assignable to the final product.

The future research aims and objectives involve linking the testbed to a standard MES control enabled by an experienced production operator and testing the production response rate, robustness, OEE, and other factors related to both of the production control options in the same scenarios. Importantly, the use of the created discrete event system will be further investigated too. This plan, however, involves certain limitations, especially in that the research and real-world production testing will require the technology to be employed in the entire manufacturing plant; such a precondition then means that the lengthy fine-tuning and commissioning may generate substantial costs. In this context, the testbed facilitates monitoring and improving the functionalities and responses to diverse errors and nonstandard situations.

## Figures and Tables

**Table 1:** The OPC UA ISO/OSI model.

Layer	Description	
7 Application	UA Application (C/S, Pub/Sub)	
6 Presentation	UA Binary	UA XML
5 Session	UA TCP UA Secure Conversation	OAP/HTTP WS-Secure Conversation
4 Transport	TCP (RFC 793)	
3 Network	IP (RFC791)	
2 Data Link	MAC (IEEE 802.3)	
1 Physical	e.g., Ethernet (IEEE 802.3)	

**Table 2:** Comparing the AAS formation options.

Category	Manually	ConfigWizard (Automated)
Developing time	very exhausting	minimized
Knowledge of the developer	demanding	straight-forward
Modifications	not featured	supported
User-friendly	dependable	click and play
Compliance with the standard	dependable	hard-wired

**Table 3:** The communication statistics.

Message Communication Type	Message Count	Average Time to Receive Proposal or Refuse Message [ms]	First Product Manufacturing Time [mm:ss]	Duration of Whole Production [mm:ss]
OPC UA LDS and OPC UA method call	5277	363	03:00	06:58
MQTT	6666	28	02:51	06:45
MQTT providers using queue	1488	130	01:35	12:16

## Data Availability

Not applicable.
